# Educational and health outcomes associated with bronchopulmonary dysplasia in 15-year-olds born preterm

**DOI:** 10.1371/journal.pone.0222286

**Published:** 2019-09-11

**Authors:** David Drummond, Alice Hadchouel, Heloise Torchin, Jean-Christophe Rozé, Catherine Arnaud, Adèle Bellino, Laure Couderc, Stéphane Marret, Marie Mittaine, Didier Pinquier, Marie Vestraete, Jessica Rousseau, Pierre-Yves Ancel, Christophe Delacourt, for the EPIPAGEADO study group

**Affiliations:** 1 Paediatric Pulmonology, University Hospital Necker-Enfants Malades, AP-HP, Paris, France; 2 Paris Descartes University, Paris, France; 3 Port Royal Hospital, AP-HP, Paris, France; 4 Department of Neonatal Medicine, Nantes University Hospital, Nantes, France; 5 CIC004, Nantes University Hospital, Nantes, France; 6 UMR1027, INSERM, Toulouse, France; 7 Paul Sabatier University, Toulouse, France; 8 Clinical Epidemiology Unit, CHU Purpan, Toulouse, France; 9 Clinical Research Unit Cochin-Necker, AP-HP, Paris, France; 10 Rouen University Hospital, Pediatric Pulmonology and Allergology, Inserm CIC-CRB 1404 Rouen, France; 11 INSERM U1245, Team 4, Perinatal Neurological Handicap and Brain Protection, IRIB, School of Medicine, Normandy University, Rouen, France and Department of Neonatal Medicine and Neuropediatrics, Rouen University Hospital, Rouen, France; 12 Paediatric Pulmonology and Allergology Department, CHU Purpan, Toulouse, France; 13 CRCM Enfants, University Hospital, Nantes, France; 14 Obstetrical, Perinatal, and Paediatric Epidemiology Team, Epidemiology and Biostatistics Sorbonne Paris Cité Research Centre (U1153), INSERM, Paris, France; 15 Clinical Research Unit, Centre for Clinical Investigation, P1419 Cochin Broca Hôtel-Dieu, APHP, Paris, France; Hopital Robert Debre, FRANCE

## Abstract

**Introduction:**

To evaluate the consequences of bronchopulmonary dysplasia (BPD) on academic outcomes and healthcare use in adolescents born very preterm.

**Methods:**

This cohort study included 15-year-old adolescents born very preterm (< 32 weeks) between 2011 and 2013, with and without BPD, and controls born full term. Data regarding academic performance, current medical follow-up, and family characteristics were collected. Multivariate logistic regression was used to quantify relationships between academic outcomes and BPD.

**Results:**

From the 1341 children included in the initial cohort, 985 adolescents were eligible and 351 included (55 preterms with a history of BPD, 249 without, and 47 controls). Among adolescents born very preterm, a history of BPD was associated with a higher risk to attend a school for children with special needs (p < 0.05) and to have repeated a grade (p = 0.01). It was also associated with an increased number of medical and paramedical consultations. A history of BPD was not associated with the parents’ employment status, family structure, or the presence of younger siblings.

**Conclusion:**

This study highlights that a history of BPD is associated with poorer academic outcomes and high healthcare use in adolescence.

## Introduction

Extreme and very preterm births are associated with cognitive impairment and lower academic outcomes in school-age children [1–3]. Bronchopulmonary dysplasia (BPD) appears in many studies to be a worsening factor, with poorer educational outcomes, regardless of the neurological prognosis [2,4–9]. A recent meta-analysis confirmed BPD as an important and independent risk factor for poor academic outcomes in school-age children born preterm [10]. It is unknown whether a history of BPD is still associated with lower academic outcomes and increased healthcare use in adolescence. Previous studies which evaluated these outcomes included adolescents born before the surfactant and antenatal steroid treatment era [11–15] and/or did not differentiate between adolescents with or without a history of BPD [11–19]. In this report, we compare the academic outcomes and healthcare use at age 15 in adolescents born very preterm who developed BPD to those of adolescents who did not develop BPD or were born full term.

## Methods

### Study design

EPIPAGE (Etude EPIdémiologique sur les Petits Ages Gestationnels) was a prospective observational population-based cohort that included all births (live births and stillbirths) and late pregnancy terminations that occurred between 22 and 32 completed weeks of gestation in all maternity wards of nine French regions in 1997[20]. A control group of individuals born at term (one in every four births at 39 or 40 weeks of gestation during 1 week in 1997) was also included. Because the study focused on the causes and consequences of prematurity, and because the control group was expected to be more homogenous than the preterm group, less children were included in the control group than in the preterm group.

At recruitment in the maternity or neonatal unit, parents were informed of the study and given written information. Oral consent was given to the medical team in charge of the study in accordance with French rules in force in 1997. The study was approved by the French Commission Nationale de l’Informatique et des Libertés (the French data protection agency).

Data for mothers, pregnancies, births, and neonatal outcomes were recorded on standardized questionnaires at each maternity and neonatal intensive care unit. Maternal data included ethnic origin, tobacco consumption during pregnancy, and causes of preterm birth. Neonatal data included gender, gestational age (determined from the last menstrual period and findings from early prenatal ultrasound scans, calculated in complete weeks), birth weight, postnatal sepsis (defined as a postnatally acquired infection treated with antibiotics for at least 7 days), necrotising enterocolitis, and bronchopulmonary dysplasia (BDP). BPD was defined as the need for supplemental oxygen and/or ventilatory support at 36 weeks of post-menstrual age. Information about the health and the development of the children was subsequently collected by questionnaires sent to the families two months after discharge, and when the child was nine months, and one, two, three, and four years old. At two years of age, a questionnaire was also sent to the child’s physician. At five years of age, the children were invited for a check-up with a physician and a psychologist at local centres in every region. At eight years of age, a questionnaire was sent to the families.

EPIPAGEADO was part of the EPIPAGE study but was restricted to the very preterm born children in four French regions: Paris, Normandie, Pays-de-la-Loire, and Midi-Pyrénées. The EPIPAGEADO (“Etude EPIdémiologique sur les Petits Ages Gestationnels- Adolescents”) study was registered at clinicaltrials.gov (NCT01424553) and approved by the ethical committee CPP Ile de France VI. The inclusion criteria were complete participation in EPIPAGE from birth, available assessment at five and/or eight years of age, coverage by national health insurance, and written parental informed consent. Inclusion took place between November 2011 and June 2013, when adolescents reached the age of 15 years.

### Data collection

Data for mothers, pregnancies, births, and neonatal outcomes were recorded on standardised questionnaires at each maternity and neonatal intensive care unit.

The assessment at 15 years of age included a questionnaire completed by the investigator with the participants and their parents (S1 and S2 Figs). Data regarding academic performance, current medical follow-up, and family characteristics were collected. The assessment also included anthropometric measures and lung function tests that were published in a previous study[21].

### Statistics

Continuous variables were compared using the Kruskal-Wallis test and categorical variables using the Chi-2 and Fisher’s exact tests. Multivariate logistic regression was used to quantify relationships between academic outcomes and BPD before and after adjustment for potential confounders identified as relevant factors from the literature (S1 Table). Retinopathy of prematurity (ROP) was not included in the main analyses as a parameter for adjustment because 26% of values were missing. However, analyses on a smaller dataset including ROP as a potential confounder were conducted (S2 Table).

Results are reported as crude and adjusted ORs with 95% confidence intervals (CIs). Significance was set at p ≤ 0.05. Analyses were performed with SAS version 9.3 software (SAS Institute, Inc, Cary, NC).

## Results

### Population

**Fig 1:**
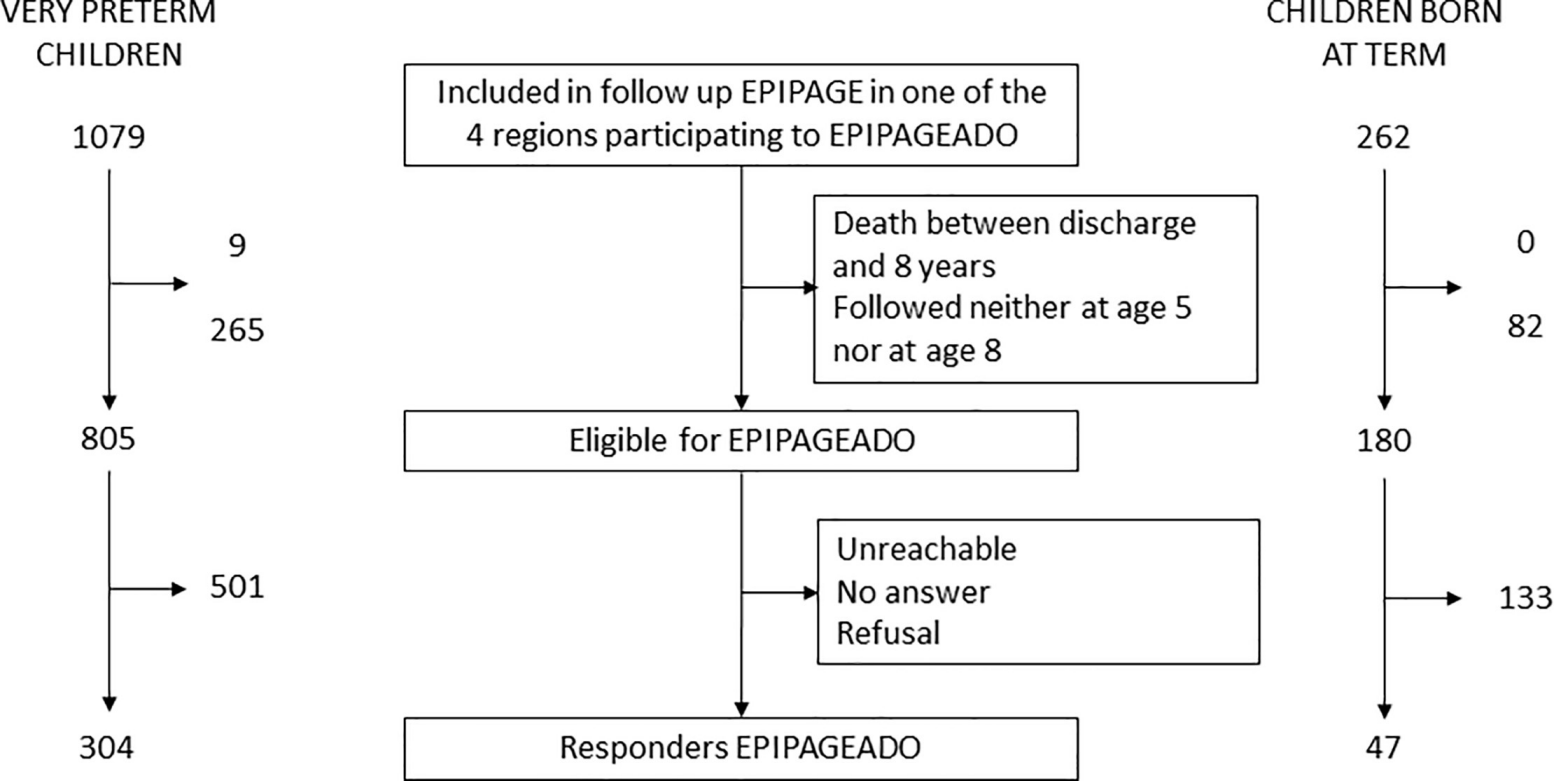
Flow-chart of the study population.

From the 1341 children included in the four French regions participating to EPIPAGEADO, 985 adolescents were eligible and 351 included (Fig 1).

The study included 304 adolescents born very preterm, 55 with and 249 without a history of BPD, and 47 controls born full term. Their characteristics are presented in Table 1.

**Table 1:** Characteristics of adolescents born very preterm with and without BPD, and controls.

	Ex-preterm with BPD n = 55 (%)	Ex-preterm without BPD n = 249 (%)	Controls n = 47 (%)	p All groups	p Preterms vs controls	p Preterms : BPD vs non BPD
Sex						
▪ Male	31 (56%)	123 (49%)	18 (38%)	0.19	0.12	0.37
▪ Female	24 (44%)	126 (51%)	29 (62%)			
Spontaneous prematurity	29/55 (53%)	130/243 (53%)	NA	NA	NA	1.00
Gestational age^a^	27 (26–29)	30 (29–31)	40 (39–40)	<0.01	<0.01	<0.01
Birth weight^a^	870 (780–1060)	1370 (1050–1640)	3430 (3210–3710)	<0.01	<0.01	<0.01
▪ < 1000 g	39 (71%)	50 (20%)	0 (0%)	<0.01	<0.01	<0.01
▪ 1000–1499 g	13 (24%)	102 (41%)	0 (0%)			
▪ ≥ 1500 g	3 (5%)	97 (39%)	47 (100%)			
						
Small for gestational age	6 (11%)	18 (7%)	0 (0%)	0.08	0.06	0.41
Apgar score at 1 min ≤ 3	22/54 (41%)	47/233 (20%)	1 (2%)	<0.01	<0.01	<0.01
Patent ductus arteriosus	9 (16%)	2 (1%)	NA	NA	NA	<0.01
Intraventricular hemorrhage (all grades)	25 (45%)	50 (20%)	NA	NA	NA	<0.01
Periventricular leukomalacia	8/53 (15%)	21/234 (9%)	NA	NA	NA	0.21
Necrotising enterocolitis	3/55 (5%)	4/248 (2%)	NA	NA	NA	0.12
Retinopathy of prematurity	11/45 (24%)	12/178 (7%)	NA	NA	NA	<0.01
Confirmed maternofetal infection	6/54 (11%)	21/241 (9%)	NA	NA	NA	0.60
≥ 1 Sepsis	39/54 (72%)	60/248 (24%)	NA	NA	NA	<0.01
Post-natal steroids	44/55 (80%)	45/247 (18%)	NA	NA	NA	<0.01
SES of family						
▪ Professional	8/55 (15%)	62/247 (25%)	20/47 (43%)	0.02	<0.01	0.06
▪ Intermediate	17/55 (31%)	84/247 (34%)	12/47 (26%)			
▪ Administrative/public service, self-employed, student	21/55 (38%)	57/247 (23%)	10/47 (21%)			
▪ Shop-assistant, service worker	3/55 (5%)	28/247 (11%)	2/47 (4%)			
▪ Manual worker or unemployed	6/55 (11%)	16/247 (6%)	3/47 (6%)			
Maternal age at birth						
▪ < 25	11/55 (20%)	27/244 (11%)	8/47 (17%)	0.04	0.13	0.10
▪ 25–34	29/55 (53%)	162/244 (66%)	34/47 (72%)			
▪ ≥ 35	15/55 (27%)	55/244 (23%)	5/47 (11%)			
Parity at birth						
▪ 0	30/55 (55%)	150/24 7 (61%)	19/47 (40%)	<0.01	<0.01	0.12
▪ 1–2	19/55 (35%)	87/247 (35%)	28/47 (60%)			
▪ ≥ 3	6/55 (11%)	10/247 (4%)	0 (0%)			
Maternal level of education						
▪ University	20/54 (37%)	106/245 (43%)	29/47 (62%)	0.02	<0.01	0.71
▪ Secondary school 2^nd^ part	15/54 (28%)	67/245 (27%)	2/47 (4%)			
▪ Secondary school 1^st^ part	17/54 (31%)	60/245 (24%)	15/47 (32%)			
▪ Primary school or no school	2/54 (4%)	12/245 (5%)	1/47 (2%)			
Country of birth of mother						
▪ France	47/55 (85%)	202/245 (82%)	46 (98%)	0.03	<0.01	0.69
▪ Other	8/55 (15%)	43/245 (18%)	1 (2%)			

a: Results are presented as median with their interquartile 1–3.

BPD: bronchopulmonary dysplasia. SES: socioeconomic status

Compared to adolescents born very preterm, those born full-term lived in families with a higher socio-economic status, from mothers with a higher level of education and a lower level of parity, and more frequently born in France (Table 1).

Among adolescents born very preterm, those with a history of BPD were born at a lower gestational age, with a lower birth weight, had a lower Apgar score at 1 min, were more frequently diagnosed with patent ductus arteriosus, intraventricular haemorrhage of all grades, and sepsis, and were more frequently treated with post-natal steroids (Table 1).

The included adolescents belonged to families characterised by a higher socio-economic status, a higher maternal level of education, and a higher proportion of mothers born in France than those lost to follow-up (S3 and S4 Tables).

### Academic achievement

Academic achievement among adolescents born very preterm with or without a history of BPD, and controls born full term, is shown in Table 2.

**Table 2:** Academic, health and family outcomes of adolescents born very preterm with BPD, born very preterm without BPD, and controls born full term.

	Ex-preterms with BPD (n = 55)	Ex-preterms without BPD (n = 249)	Controls (n = 47)	p All groups	p Preterms vs controls	p Preterms : BPD vs non BPD
**Academic outcomes**						
School adapted for children with special needs or specialized institution (vs ordinary school)	14/55 (25%)	15/248 (6%)	1/47 (2%)	<0.01	0.10	< 0.01
Repeated grade	23/54 (43%)	54/246 (22%)	2/47 (4%)	<0.01	<0.01	< 0.01
Personalized assistance at school	11/55 (20%)	9/247 (4%)	2/47 (4%)	<0.01	0.75	< 0.01
**Healthcare use**						
Specialist follow-up in the past 12 months	19/55 (35%)	61/249 (25%)	10/47 (21%)	0.23	0.59	0.13
Physiotherapy in the last 12 months	10/55 (18%)	47/248 (19%)	6/47 (13%)	0.60	0.42	0.89
Psychomotor therapist in the past 12 months	6/55 (11%)	2/248 (1%)	0/47 (0%)	<0.01	0.50	<0.01
Speech therapist in the past 12 months	13/55 (24%)	15/248 (6%)	2/47 (4%)	<0.01	0.40	<0.01
Psychologist or psychiatrist in the past 12 months	15/55 (27%)	33/248 (13%)	2/47 (4%)	<0.01	0.04	0.01
≥ 1 hospital admission in the past 5 years	12/55 (22%)	44/248 (18%)	9/47 (19%)	0.69	1.00	0.48
**Family characteristics**						
Two-parent family (vs single-parent family)	46/55 (84%)	199/248 (80%)	40/46 (87%)	0.51	0.41	0.55
Younger siblings	26/55 (47%)	104/248 (42%)	27/47 (59%)	0.10	0.06	0.47
Mother employment	45/55 (82%)	195/248 (79%)	42/47 (91%)	0.13	0.63	0.60
Father employment	46/53 (87%)	214/244 (88%)	42/47 (91%)	0.75	0.07	0.86

BPD: bronchopulmonary dysplasia

Significantly more adolescents born very preterm had repeated a grade (i.e., were not considered ready to graduate by their teachers and had to repeat a year at the same grade) than controls born full preterm.

Among adolescents born very preterm, significantly more adolescents with a history of BPD attended a school adapted for children with special needs, had repeated a grade, and needed personalized assistance at school than adolescents without a history of BPD (Table 2). After adjustment for sex, gestational age, small for gestational age, intraventricular hemorrhage, periventricular leukomalacia, necrotising enterocolitis, late-onset sepsis, post-natal steroids, family structure, maternal level of education and socio-economic status, a history of BPD was still associated with attending a school adapted for children with special needs and repeating a grade (Table 3).

**Table 3:** Comparison of academic, health and family outcomes of adolescents born very preterm with and without BPD.

	Ex-preterms with BPD (n = 55)	Ex-preterms without BPD (n = 249)	Crude OR (95% CI)	Crude p value	Adjusted OR (95% CI)	Adjusted p value
**Academic outcomes**^a^						
School adapted for children with special needs or specialized institution (vs ordinary school)	14/55 (25%)	15/248 (6%)	5.3 (2.4–11.8)	< 0.01	3.4 (1.0–11.6)	0.047
Repeated grade	23/54 (43%)	54/246 (22%)	2.6 (1.4–4.9)	< 0.01	2.7 (1.1–6.6)	0.03
Personalized assistance at school	11/55 (20%)	9/247 (4%)	6.6 (2.6–16.9)	< 0.01	1.7 (0.4–8.2)	0.51
**Healthcare use**^b^						
Specialist follow-up in the past 12 months	19/55 (35%)	61/249 (25%)	1.6 (0.9–3.0)	0.13	1.3 (0.6–2.7)	0.49
Physiotherapy in the last 12 months	10/55 (18%)	47/248 (19%)	0.95 (0.5–2.0)	0.89	0.6 (0.2–1.4)	0.21
Psychomotor therapist in the past 12 months	6/55 (11%)	2/248 (1%)	15.1 (3.0–77.0)	<0.01	5.5 (0.8–36.4)	0.08
Speech therapist in the past 12 months	13/55 (24%)	15/248 (6%)	4.8 (2.1–10.8)	<0.01	3.7 (1.3–10.2)	0.01
Psychologist or psychiatrist in the past 12 months	15/55 (27%)	33/248 (13%)	2.4 (1.2–4.9)	0.01	1.6 (0.7–3.8)	0.26
≥ 1 hospital admission in the past 5 years	12/55 (22%)	44/248 (18%)	1.3 (0.6–2.6)	0.48	1.0 (0.4–2.3)	0.93
**Family characteristics**						
Two-parent family (vs single-parent family)^c^	46/55 (84%)	197/248 (79%)	0.8 (0.4–1.7)	0.56	1.0 (0.4–2.4)	0.98
Younger siblings^c^	26/55 (47%)	104/248 (42%)	1.2 (0.7–2.2)	0.47	1.3 (0.7–2.6)	0.44
Mother employment^d^	45/55 (82%)	195/248 (79%)	1.2 (0.6–2.6)	0.60	1.2 (0.5–3.1)	0.72
Father employment^d^	46/53 (87%)	214/244 (88%)	0.9 (0.4–2.2)	0.86	0.7 (0.2–2.2)	0.49

a: Adjusted OR and p value are adjusted for sex, gestational age, small for gestational age, intraventricular hemorrhage, periventricular leukomalacia, sepsis, necrotising enterocolitis, post-natal steroids, family structure, maternal level of education and socioeconomic status.

b: Adjusted OR and p value are adjusted for sex, gestational age, small for gestational age, intraventricular hemorrhage, periventricular leukomalacia and necrotising enterocolitis.

c: Adjusted OR and p value are adjusted for sex, gestational age, small for gestational age, intraventricular hemorrhage, periventricular leukomalacia and specialist follow-up.

d: Adjusted OR and p value are adjusted for sex, gestational age, small for gestational age, intraventricular hemorrhage, periventricular leukomalacia, specialist follow-up, family structure, number of siblings, and socioeconomic status.

BPD: bronchopulmonary dysplasia.

### Healthcare use

Significantly more adolescents born very preterm (with and without BPD) had been under the care of a psychologist of a psychiatrist in the previous 12 months than controls born full preterm (Table 2). By contrast, the proportion of adolescents who had been under the care of a medical specialist, a psychomotor therapist, a speech therapist in the previous 12 months, or who required a hospital admission in the past 5 years did not differ significantly between adolescents born very preterm and controls born full term.

Among adolescents born very preterm, those with a history of BPD were more likely to have been under the care of a psychomotor therapist, a speech therapist, and a psychologist or psychiatrist in the previous 12 months (Table 2). However, after adjustment for sex, gestational age, small for gestational age, intraventricular haemorrhage, periventricular leukomalacia, and necrotising enterocolitis, a history of BPD remained only associated with visiting a speech therapist in the previous 12 months (Table 3).

Although the overall use of a specialist or physiotherapist was identical between adolescents with or without a history of BPD, a detailed analysis by specialty showed that BPD infants used more respiratory care resources (S5 Table)

### Family characteristics

Neither a history of prematurity nor a history of BPD were associated with the parents’ employment status, family structure, or the presence of younger siblings (Table 2 and S6 Table).

## Discussion

This cohort study shows that the burden of BPD on health and educational services extends after childhood to 15-year-old adolescents, despite the progress made in the 1990’s in perinatal management of very preterm infants, with the generalization of surfactant use and antenatal steroid treatment.

Concerning academic achievement, one quarter of the adolescents with BPD included in our cohort attended a school adapted for children with special needs or a specialised institution and 40% had repeated a grade. These results are in accordance with previous studies which consistently found lower academic achievement among younger children who had BPD than among their peers. A meta-analysis identified BPD as the most important risk factor for poor academic outcomes in preterm children born in the antenatal steroid and surfactant era, accounting for 44% of the variance in academic performance [10]. Our study provides new insight showing that this finding extends to adolescence.

The negative impact of BPD on academic achievement does not appear to be attributable to the persistence of respiratory morbidity associated with this disease throughout childhood and adolescence. Indeed, no hospital admission for respiratory conditions in adolescents with BPD was recorded during the previous 12 months in our series. Instead, our data indicate an association between BPD and neurological morbidity, with more adolescents with BPD requiring consultations with psychomotor therapists, speech therapists, and psychologists or psychiatrists.

The family characteristics of adolescent born very preterm with BPD are more reassuring. In this study, being born very preterm with BPD was not associated with lower parental employment rates than in the French general population [22] and did not prevent families from having other children.

The main limitation of this cohort study, as in other longitudinal studies, was the high proportion of adolescents lost to follow-up (64%). Similar studies that further investigated children who were initially lost to follow-up report a worse outcome for such dropout groups [23,24]. It is likely that this is also true for our cohort. The rate of adolescents born full term who repeated a grade that we observed (4%) was much lower compared to the rates of 23.7% and 28% recorded in two surveys conducted among French 15-year-old adolescents during the same year [25]. This lower rate is explained by the higher socio-economic status and higher maternal level of education in the families of included adolescents, two factors associated with higher academic achievement. Because adolescents born very preterm also belonged to families with higher socio-economic status and higher level of education than those who were lost to follow-up, our results may underestimate the impact of prematurity and BPD on academic outcomes. A second limitation was that the evaluation of academic achievements was solely based on family interviews and not on objective assessments of different educational dimensions. Finally, BPD was defined by the need for supplemental oxygen at 36 weeks of post-menstrual age as it was common practice in 1997, before Walsh et al. published their oxygen reduction challenge in 2003 [26]. The limitation of the definition used in our study is that the need for oxygen was determined by individual physicians, while divergent practices regarding oxygen saturation exist among neonatologists.

In conclusion, this study highlights that a history of BPD is associated with poorer academic outcomes and important healthcare use in adolescence. The magnitude of these effects may be underestimated due to a non-response bias.

## Supporting information

S1 Fig Questionnaire (in French) used to collect data from 15 years-old patients and their parents participating in the EPIPAGEADO study. (PDF)

S2 Fig English translation of the questions used for the study. (PDF)

S1 Table Characteristics used for adjustment for each outcome studied. (PDF)

S2 Table Comparison of academic and health outcomes of adolescents born very preterm with and without BPD, adjusted on parameters presented in Table 3 and retinopathy of prematurity. (PDF)

S3 Table Characteristics of adolescents born very preterm included and lost to follow-up. (PDF)

S4 Table Characteristics of adolescents control (full term) included and lost to follow-up. (PDF)

S5 Table Healthcare use in adolescents born very preterm with and without BPD. (PDF)

S6 Table Family characteristics of adolescents born very preterm with and without BPD. (PDF)
