# Supplementary material for: Educational and health outcomes associated with bronchopulmonary dysplasia in 15-year-olds born preterm
Source: PLoS One. 2019 Sep 11;14(9):e0222286. doi: 10.1371/journal.pone.0222286 (PMC6738652; doi:10.1371/journal.pone.0222286)
Supplement: S1 Fig — (PDF) [file pone.0222286.s001.pdf]

# Inclusion

## Inclusion

# EPIPAGEADO

### Caractéristiques générales

Centre _______________________________________ Centre N° |__|__|
_____________________________________________
_____________________________________________
_____________________________________________
_____________________________________________
_____________________________________________
_________

Investigateur ____________________________________________________________________________
______________________________

Identifiant d'Inserm |__|__|__|__|__|__|__|__|__|__|

Numéro du Patient |__|__|__|

Initial du Nom |__| Initial du Prénom |__|

Référence Patient |__|__|__|__|__|__|__|

Sexe : ○ Masculin ○ Féminin

Poids |__|__|__| , |__| Kg Taille |__|__|__| Cm

**En cas de l'absence du jour de naissance, mettez 01 à la place du jour.**

Date de naissance |__|__|/|__|__|/|__|__|__|__|

**Pour l'enquête génétique :**

Pays d'origine de la Mère ____________________________________________________________________
_______________________________________

Pays d'origine du Père ______________________________________________________________________
_____________________________________

**Prématurité :**

○ Prématurés sans dysplasie bronchopulmonaire (DBP)
○ Prématurés avec dysplasie bronchopulmonaire
○ Témoins (nés de 39 à 40 SA)
○ Non connue

### Critères d'Inclusion et de Non-Inclusion

# Critères d'inclusion

**1. Les grands prématurés et les enfants témoins nés à terme participant au suivi de cohorte EPIPAGE depuis leur naissance.**

**2. Consentement éclairé des parents**

**3. Sécurité sociale (bénéficiaire ou ayant droit)**

## Critères de non inclusion

**1. Les enfants ayant un déficit neurologique sévère**

**Tous les critères sont-ils vérifiés ?** ○ Oui ○ Non

Si non, préciser lesquels : ____________________________________________________________
__________________________________________________________________________________
__________________________________________________________________________________
__________________________________________________________________________________
____________________________________________

**Voulez-vous inclure le patient ?** ○ Oui ○ Non

Date de l'Inclusion : |__|__|/|__|__|/|__|__|__|__|

**Veuillez enregistrer avant de fermer le formulaire**

# Scolarité, Histoire maladie, vie famille

## Situation scolaire actuelle

Pour l'année scolaire en cours, es-tu inscrit dans un établissement scolaire ou d'apprentissage ? ◯ Oui ◯ Non

Si OUI, dans quel type d'établissement es-tu scolarisé(e) :
◯ 1. au collège en enseignement général
◯ 2. au collège en SEGPA
◯ 3. au collège en UPI ou ULIS (unité pour l'inclusion scolaire)
◯ 4. dans un EREA (établissement régional d'enseignement adapté)
◯ 5. au lycée en classe d'enseignement général
◯ 6. au lycée en classe d'enseignement technologique
◯ 7. au lycée en classe d'enseignement professionnel
◯ 8. au lycée en UPI ou ULIS (unité pour l'inclusion scolaire)
◯ 9. en institution spécialisée
◯ 10. en hôpital de jour
◯ 11. centre de formation d'apprentis
◯ 12. dans un autre lieu

Si autre, préciser : ____________________________________________________________________
_____________________________________________________________________________
____________________________________________

### *Si NON*

Suis-tu des cours à distance par correspondance (CNED) ? ◯ Oui ◯ Non

Es-tu instruit(e) à ton domicile par tes parents ou un professeur particulier ? ◯ Oui ◯ Non

### *Si tu es au collège ou au lycée :*

De quel type d'établissement s'agit-il ? ◯ Public ◯ Privé

◯ Internat ◯ Externat

Dans quelle classe es-tu :
◯ en 6ème
◯ en 5ème
◯ en 4ème
◯ en 3ème
◯ en première année de CAP
◯ en deuxième année de CAP
◯ en 2nde
◯ en 1ère
◯ autre

Dans ce cas précisez ________________________________________________________________
______________________________

Depuis que tu vas à l'école, as-tu déjà redoublé une classe ?
◯ Non
◯ Oui, une classe
◯ Oui, plus d'une classe

Si oui, laquelle ou lesquelles: _________________________________________________________
_____________________________________________________________________________

_ _ _ _ _ _ _ _ _ _ _ _ _ _ _ _ _ _ _ _ _ _ _ _ _ _ _ _ _ _ _ _ _ _ _ _ _ _ _ _ _ _ _ _ _ _ _ _ _ _ _ _ _ _ _ _ _ _ _

Suis-tu des cours de soutien scolaire en dehors du collège ou du lycée ? ○ Oui ○ Non

A l'école, bénéficies-tu d'une aide personnalisée, c'est-à-dire quelqu'un qui est présent pour t'aider dans tes déplacements ou pour certaines activités particulières ? ○ Oui ○ Non

**Veuillez enregistrer avant de fermer le formulaire**

## Histoire médicale

### Page1

Prise de traitements réguliers dans les 12 derniers mois (supérieure à 3 mois par an) : ○ Oui ○ Non

**Si Oui, le(les)quel(s) :**

- ☐ corticothérapie inhalée
- ☐ Traitement anti-reflux
- ☐ Traitement anti-épileptique
- ☐ Bronchodilatateur
- ☐ Traitement anti-allergique
- ☐ Traitement anti leucotriènes
- ☐ Autre

Si corticothérapie, précisez la molécule : _ _ _ _ _ _ _ _ _ _ _ _ _ _ _ _ _ _ _ _ _ _ _ _ _ _ _ _ _ _ _ _ _ _ _ _ _ _ _ _ _ _ _ _ _ _ _ _ _ _ _

Si corticothérapie, précisez la dose quotidienne administrée : |__|__|__| , |__| µg

Si bronchodilatateur, précisez :
☐ B2 Longue action ☐ B2 Courte action

Si autre traitement , précisez : _ _ _ _ _ _ _ _ _ _ _ _ _ _ _ _ _ _ _ _ _ _ _ _ _ _ _ _ _ _ _ _ _ _ _ _ _ _ _ _ _ _ _ _ _ _ _ _ _ _ _ _ _ _ _ _ _ _ _ _ _ _ _ _ _ _ _ _ _ _ _ _ _ _ _ _ _ _ _ _ _ _ _ _ _ _ _ _ _ _ _ _ _ _ _ _ _ _ _ _ _ _ _ _ _ _ _ _ _ _ _ _ _ _ _ _ _ _ _ _ _ _ _ _ _ _ _ _ _ _ _ _ _ _ _ _ _ _ _ _ _ _ _ _ _ _

Si anti leucotriènes, précisez :
○ Longue action ○ Courte action

Suivi médical spécialisé régulier (dans les 12 derniers mois) : ○ Oui ○ Non

**Si Oui, spécialité(s) :**

- ☐ pneumologie
- ☐ gastro entérologie
- ☐ neurologie
- ☐ orthopédie
- ☐ autre

Si autre, précisez : _ _ _ _ _ _ _ _ _ _ _ _ _ _ _ _ _ _ _ _ _ _ _ _ _ _ _ _ _ _ _

### Page2

Au cours des 12 derniers mois, as-tu eu des séances de kinésithérapie ? ○ Oui ○ Non

Si Oui, précisez : ☐ Respiratoire ☐ Motrice

Des séances de psychomotricité ? ○ Oui ○ Non

Des séances d'orthophonie ? ○ Oui ○ Non

Au cours des 12 derniers mois, es-tu allé(e) chez un
psychologue, un psychiatre, dans un CMP ou un CMPP ?

○ Non
○ Oui, une seule fois dans l'année
○ Oui, plusieurs fois
○ Oui, chaque semaine ou presque

Nombre d'hospitalisations au cours des 5 dernières années : |__|__|

Nombre d'hospitalisations pour cause respiratoire au cours des 5 dernières années : |__|__|

Nombre d'hospitalisations pour cause respiratoire depuis la sortie de néonatologie : |__|__|

Page3

Antécédents d'asthme ou allergie chez les parents ou la fratrie : ○ Oui ○ Non

Asthme
☐ Mère ☐ Père ☐ Fratrie ☐ Non applicaple

Allergie respiratoire
☐ Mère ☐ Père ☐ Fratrie ☐ Non applicable

Eczéma atopique
☐ Mère ☐ Père ☐ Fratrie ☐ Non applicable

## Tabagisme passif de l'enfant :

La maman fumait-elle pendant la grossesse ? ○ Oui ○ Non

Si OUI : Nombre moyen de cigarette par jour : |__|__|

Un adulte fumait-il à la maison lorsque l'enfant avait entre 0 et 2 ans ? ○ Oui ○ Non

Si Oui, précisez : ☐ Père ☐ Mère ☐ Autre

Et comment ? ○ En présence de l'enfant ○ En dehors de la présence de l'enfant

Un adulte fumait-il à la maison dans les 12 derniers mois ? ○ Oui ○ Non

Si Oui, précisez : ☐ Père ☐ Mère ☐ Autre

**Veuillez enregistrer avant de fermer le formulaire**

# Mode de vie familiale

Page1

**Les questions suivantes sont à poser en dehors de la présence des parents**

Où vis-tu la plupart du temps ? (en dehors
de l'internat si tu es pensionnaire) ?

○ avec ta mère et ton père
○ avec ta mère
○ avec ton père

○ de manière alternée chez l'un ou l'autre
○ chez un autre membre de la famille
○ dans une famille d'accueil
○ dans une institution (foyer, IME, autre à préciser ...)
○ ...........

Si tu ne vis pas chez ta mère, à quelle fréquence vois-tu ta mère ?

○ au moins 2 fois par semaine
○ 1 fois par semaine
○ au moins une fois par mois
○ lors des congés scolaires
○ autre situation
○ mère décédée

Si tu ne vis pas chez ton père, à quelle fréquence vois-tu ton père ?

○ au moins 2 fois par semaine
○ 1 fois par semaine
○ au moins une fois par mois
○ lors des congés scolaires
○ autre situation
○ père décédé

Nombre de frères ou soeurs plus âgés : |__|__|

Nombre de frères ou soeurs plus jeunes : |__|__|

Nombre de demi-frères ou soeurs plus âgés : |__|__|

Nombre de demi-frères ou soeurs plus jeunes : |__|__|

Ta mère a-t-elle un emploi actuellement ?

○ OUI, temps plein
○ OUI, temps partiel
○ NON, elle cherche du travail
○ NON, elle s'occupe de la maison
○ NON, autre raison

Ton père a-t-il un emploi actuellement ?

○ OUI, temps plein
○ OUI, temps partiel
○ NON, il cherche du travail
○ NON, il s'occupe de la maison
○ NON, autre raison

Page2

# Tabagisme actif

As-tu déjà essayé de fumer, ne serait-ce qu'une seule cigarette ? ○ Oui ○ Non

## Si Oui :

à quel âge as-tu fumé ta 1ère cigarette ? |__|__| ans

As-tu fumé au moins une cigarette au cours du dernier mois ? ○ Oui ○ Non

As-tu fumé au moins une cigarette au cours de la dernière semaine ? ○ Oui ○ Non

Fumes-tu au moins une cigarette par jour ? ○ Oui ○ Non

Eprouves-tu le besoin de fumer de plus en plus, au fil du temps ? ○ Oui ○ Non

As-tu réduit tes activités sportives parce que tu fumais ? ○ Oui ○ Non

**Veuillez enregistrer avant de fermer le formulaire**

# Questionnaire ISAAC

## Sifflements respiratoires

**<u>QUESTIONNAIRE SUR LES SIFFLEMENTS RESPIRATOIRE ET L'ASTHME</u>**

1- Depuis votre naissance, avez vous déjà eu au moins 1 fois des sifflements respiratoires, ou des sifflements au niveau de la poitrine ? ○ Oui ○ Non

**<u>Si vous avez répondu non à la question 1, veuillez directement passer à la question 6</u>**

2- Avez vous déjà présenté des sifflements respiratoires ou des sifflements dans la poitrine durant les 12 derniers mois ? ○ Oui ○ Non

**<u>Si vous avez répondu non à la question 2, veuillez directement passer à la question 6</u>**

3- Combien d'épisodes de sifflements respiratoires, avez-vous eu durant les 12 derniers mois ?

○ 1 à 3
○ 4 à 12
○ Plus de 12

4- Durant les 12 derniers mois, combien de fois en moyenne, avez vous été réveillé la nuit pour cause de sifflements ?

○ Jamais réveillé pour cause de sifflements
○ Moins d'une fois par semaine
○ Une fois ou plus d'une fois par semaine

5- Durant les 12 derniers mois, vos sifflements ont-ils été 1 fois suffisamment graves pour limiter vos paroles à seulement 1 ou 2 mots entre chaque respiration ?
○ Oui ○ Non

6- Avez vous déjà eu de l'asthme depuis votre naissance ? ○ Oui ○ Non

7- Durant les 12 derniers mois, avez vous eu des sifflements dans la poitrine pendant ou après les efforts physiques ? ○ Oui ○ Non

8- Durant les 12 derniers mois, avez vous eu une toux sèche en pleine nuit, en dehors d'une période de rhume ou de bronchite ? ○ Oui ○ Non

**Veuillez enregistrer avant de fermer le formulaire**

## Questions sur les Rhinites

**Toutes ces questions concernent des problèmes en dehors d'une grippe ou d'un rhume.**

1- Depuis votre naissance, avez-vous déjà eu des problèmes d'éternuements, de nez qui coule ou de nez bouché en dehors des périodes de rhume ou grippe ? ○ Oui ○ Non

**Si vous avez répondu non à la question 1, veuillez directement passer à la question 6 à la page suivante**

2- Durant les 12 derniers mois, avez-vous déjà eu des problèmes d'éternuements, de nez qui coule ou de nez bouché en dehors des périodes de rhume ou grippe ? ○ Oui ○ Non

**Si vous avez répondu non à la question 2, veuillez directement passer à la question 6 à la page suivante**

3- Durant ces 12 derniers mois, est ce que ces problèmes de nez ont été associés à des yeux qui piquent ou larmoyant ? ○ Oui ○ Non

4- Durant ces 12 derniers mois, durant quel(s) mois votre problème de nez est-il apparu ?

- ☐ Janvier
- ☐ Février
- ☐ Mars
- ☐ Avril
- ☐ Mai
- ☐ Juin
- ☐ Juillet
- ☐ Août
- ☐ Septembre
- ☐ Octobre
- ☐ Novembre
- ☐ Décembre

5- Durant les 12 derniers mois, est ce que ces problèmes de nez ont interféré avec vos activités journalières ?

- ○ Pas du tout
- ○ Un peu
- ○ Modérément
- ○ Beaucoup

6- Depuis votre naissance, avez-vous déjà eu le rhume des foins ? ○ Oui ○ Non

**Veuillez enregistrer avant de fermer le formulaire**

# Examens

## Examens Complémentaires

Date de l'examen |__|__|/|__|__|/|__|__|__|__|

Commentaires : ____________________________________________________________
________________________________________________________________________
________________________________________________________________________
_______________

Poids |__|__|__| , |__| Kg

Taille |__|__|__| , |__| Cm

### Pré-bronchodilatateur

Test complet ○ Oui ○ Non

Si non, précisez : __________________________________________________________
________________________________________________________________________

CV (Capacité vitale lente) |__| , |__|__| L

CRF pléthysmo |__| , |__|__| L

CPT |__| , |__|__| L

CVF (Capacité vitale forcée lente) |__| , |__|__| L

VEMS |__| , |__|__| L

DEM50 |__| , |__|__| L/S

DEM25_75 |__| , |__|__|

FENO 50 ml/s |__|__| ppb

Volume alvéolaire |__| , |__|__| L

KCO |__|__| , |__|__| mmol.min-1 .kPa-1 .L-1

DLCO |__|__| , |__|__| mmol.min -1 .kPa-1

DLNO |__|__| , |__|__|

### Post 400 µg salbutamol

Test complet ○ Oui ○ Non

Si non, précisez : __________________________________________________________
________________________________________________________________________

CRF pléthysmo |__| , |__|__| L

CVF (Capacité vitale forcée lente) |__| , |__|__| L

VEMS |__| , |__|__| L

DEM50 |__| , |__|__| L/S

DEM25_75 |__| , |__|__|

### Test Exercice et salivaire

Test excercice complet ○ Oui ○ Non

Si non, précisez : _______________________________
____________________________________________

_ _ _ _ _ _ _ _ _ _ _ _ _ _ _ _ _ _ _ _ _ _ _ _ _ _ _ _ _ _ _ _ _ _ _ _ _ _ _ _ _ _
_ _ _ _ _ _ _ _ _ _ _ _ _ _ _ _ _ _ _ _ _ _ _ _ _ _ _ _ _ _ _ _ _ _ _ _ _ _ _ _

| | |
|---|---|
| VO2 max \|__\|__\| , \|__\| ml / Kg / min | VE repos \|__\|__\|__\| , \|__\| L/min |
| VO2 au seuil ventilatoire \|__\|__\| , \|__\| ml / Kg / min | VE Max \|__\|__\|__\| , \|__\| L/min |
| | VE Seuil \|__\|__\|__\| , \|__\| L/min |
| Réserve ventilatoire au pic (%) \|__\|__\| , \|__\| | VE max / VO2 max \|__\|__\| , \|__\| |
| Réserve ventilatoire au seuil (%) \|__\|__\| , \|__\| | VE max / VCO2 max \|__\|__\| , \|__\| |
| Fc repos \|__\|__\|__\| min-1 | |
| Fc max \|__\|__\|__\| min-1 | |
| Fc seuil \|__\|__\|__\| min-1 | |
| FR max \|__\|__\| min-1 | SaO2 repos (%) \|__\|__\|__\| |
| VT max \|__\| , \|__\|__\|__\| L | SaO2 pic (%) \|__\|__\|__\| |
| PMA (watt au max) \|__\|__\|__\| | |
| Prélèvement salivaire ○ oui ○ non | |

# EI

## Nouvel EI

N° |__|__|

Nature de l'évènement : ________________________________________________________________________________________________________________________________________________

Date de survenue (durant l'étude) : |__|__|/|__|__|/|__|__|__|__|

En cours à la fin de l'étude : ◯ Non ◯ Oui

Si Non, date de disparition : |__|__|/|__|__|/|__|__|__|__|

Durée (si évènement < 24h) : |__|__| Heures Minutes |__|__| Minutes

Présence avant la recherche ? Si oui, son évolution :
◯ Non
◯ Oui, mais en régression
◯ Oui, mais stable
◯ Oui, mais en progression

Intensité :
◯ Légère
◯ Modérée
◯ Sévère

Fréquence :
◯ Unique
◯ Intermittente
◯ Constante

Imputabilité à l'étude :
◯ Paraissant exclue
◯ Douteuse
◯ Probable
◯ Non évaluable

Traitement symptomatique : ◯ Non ◯ Oui

Autre étiologie envisagée : ◯ non ◯ oui

Si oui, préciser : ________________________________________________________________________________________________________________________________________________

# EIG

## Formulaire EIG : Fiche Investigateur

**FORMULAIRE DE DECLARATION D'UN EVENEMENT INDESIRABLE GRAVE (EIG) SURVENANT AU COURS D'UNE RECHERCHE BIOMEDICALE NE PORTANT PAS SUR UN PRODUIT MENTIONNE A L'ARTICLE L.5311-1 DU CODE DE LA SANTE PUBLIQUE**

**ASSISTANCE PUBLIQUE - HÔPITAUX DE PARIS** **DIRC Ile-de-France**

| Partie réservée au Promoteur : ne pas remplir |
|---|
| **\|__\|__\| - \|__\|__\| - DRCD - \|__\|__\|__\|__\| - \|__\|__\|__\|__\|** |

**Cette fiche doit être retournée dûment complétée au DRCD par fax : +33 (0)1 44 84 17 99 (à l'attention de Shohreh AZIMI).**

**Veuillez cliquer ici pour imprimer le formulaire EIG pré-rempli**

**Date de notification :** |__|__|/|__|__|/|__|__|__|__|

Notification :

◯ Déclaration Initiale

◯ Suivi d'EIG déclaré

Numéro de Suivi _ _ _ _ _ _ _ _ _ _ _ _ _ _ _ _ _ _ _ _ _ _ _ _ _ _ _ _ _ _ _ _ _ _ _ _ _ _ _ _ _ _ _ _ _ _ _ _ _ _ _ _ _ _ _ _ _ _ _ _ _ _ _ _ _ _ _ _ _ _ _ _ _ _ _ _ _ _ _ _ _

Code de la Recherche : P 100117

N° IDRCB : 2010-A01218-31

| Titre de la recherche biomédicale |
|---|
| **EPIPAGEADO : Devenir respiratoire à l'adolescence des grands prématurés de la cohorte EPIPAGE** |

| Centre |
|---|
| **Nom et adresse du centre :** _ _ _ _ _ _ _ _ _ _ _ _ _ _ _ _ _ _ _ _ _ _ _ _ _ _ _ _ _ _ _ _ _ _ _ _ _ _ _ _ _ _ _ _ _ _ _ _ _ _ _ |
| **Centre N° :** \|__\| |
| **Investigateur :** _ _ _ _ _ _ _ _ _ _ _ _ _ _ _ _ _ _ _ _ _ _ _ _ _ _ _ _ _ _ _ _ _ _ _ _ _ _ _ _ _ _ _ _ _ _ _ _ _ _ _ _ _ _ _ _ _ _ _ _ _ _ _ _ _ _ _ _ _ _ _ _ _ _ _ _ _ _ _ _ _ _ _ _ _ _ _ _ _ _ _ _ _ _ _ _ _ _ _ _ _ _ _ _ _ |

| Patient | |
|---|---|
| Numéro du Patient \|__\|__\|__\| | |
| Identification du patient \|__\|__\|__\|__\|__\|__\| | |
| Nom : \|__\| | Prénom : \|__\| |
| Sexe : ◯ Masculin ◯ Féminin | |

Né(e) le : |__|__|/|__|__|/|__|__|__|__| Age : |__|__| ans

Poids (kg) : |__|__|__| Kg

Taille (cm) : |__|__|__| Cm

Antécédents (allergie, insuffisance rénale...) : ________________________________________________________________

Inclus le : |__|__|/|__|__|/|__|__|__|__|

Prématurité :
○ Prématurés sans dysplasie bronchopulmonaire (DBP)
○ Prématurés avec dysplasie bronchopulmonaire
○ Témoins (nés de 39 à 40 SA)

## Description

Evènement indésirable grave :
○ Décès
○ Mise en jeu du pronostic vital
○ Nécessite ou prolonge l'hospitalisation
○ Incapacité ou invalidité
○ Anomalie congénitale
○ Autre(s) critère(s) médicalement significatif(s)

Hospitalisation en cours ○ Oui ○ Non

Si pas en cours, hospitalisation du |__|__|/|__|__|/|__|__|__|__|

au |__|__|/|__|__|/|__|__|__|__|

Si "Autre", préciser : ____________________________________________________________

Intensité :
○ Légere
○ Modérée
○ Sévère

Date de survenue : |__|__|/|__|__|/|__|__|__|__|

Heure de survenue : |__|__| : |__|__|

Description complète de l'évènement indésirable (diagnostic retenu, localisation anatomique, critères permettant de considérer l'évènement comme grave) : ____________________________________________________________

Délai de survenue : ______________________________

Evolution : ○ Oui ○ Non

Si oui, préciser : ____________________________________________________________

Date de disparition : |__|__|/|__|__|/|__|__|__|__|

Heure de disparition : |__|__| : |__|__|

## Tts concomitants

**Médicament(s) concomitant(s) (à l'exclusion de ceux utilisés pour traiter l'évènement indésirable) :**

| Nom | Voie | Dose/24h | Début | En cours | Fin | Indication | Causalité |
|---|---|---|---|---|---|---|---|
| ... | ... | ... | ... | ... | ... | ... | ... |
| ... | ... | ... | ... | ... | ... | ... | ... |
| ... | ... | ... | ... | ... | ... | ... | ... |

## DM concomitant

**Description du dispositif médical (DM) concomitant**

### Dispositif médical

Nature du dispositif médical impliqué : ____________________________________________________________________________________________________

Modèle/type/référence : ____________________________________________________________________________________________________

N° de série et/ou de lot : _______________________________________________________

Nom du fournisseur : _____________________

Nom du fabricant : ____________________________________________________________________________________________________

Dispositif médical stérile : ◯ Oui ◯ Non

Si oui, date de stérilisation : |__|__|/|__|__|/|__|__|__|__|

Date de péremption : |__|__|/|__|__|/|__|__|__|__|

Causalité :
◯ Probable
◯ Possible
◯ Non liée
◯ Inconnue

## Conclusion

Autre(s) étiologie(s) envisagée(s) : ◯ Oui ◯ Non

Si oui, préciser : ____________________________________________________________________________________________________

Examens complémentaires réalisés : ◯ Oui ◯ Non

Si oui, préciser sa date, sa nature et son résultat : ____________________________________________________________________________________________________

**Selon l'Investigateur, l'évènement indésirable semble plutôt lié :**
◯ à un dispositif médical posé
◯ à un (ou plusieurs) médicament(s) administré(s)
◯ A une maladie intercurrente
◯ Aux procédures de la recherche biomédicale
◯ A la progression de la maladie
◯ Autre

Si lié a un (ou plusieurs) médicament(s) administré(s), précisez : ____________________________________________________________________________________________________________________________________________________________________________________________________________________________________________________

Si "Autre", préciser : _____________________________________________________________
