# Supplementary material for: Educational and health outcomes associated with bronchopulmonary dysplasia in 15-year-olds born preterm
Source: PLoS One. 2019 Sep 11;14(9):e0222286. doi: 10.1371/journal.pone.0222286 (PMC6738652; doi:10.1371/journal.pone.0222286)
Supplement: S2 Fig — (PDF) [file pone.0222286.s002.pdf]

Supplementary Figure 1

English version of the questions asked regarding school situation, medical history and family lifestyle, from the questionnaire of the EPIPAGEADO study

1. Current school situation

1. For the current school year, are you enrolled in a school or apprenticeship? Yes No

2. If yes, what type of school are you in?
   - Regular school
   - School for children with special needs

3. If no,
   - Do you follow distance correspondence courses? Yes No
   - Are you educated at home by your parents or a private teacher? Yes No

4. If you are in a regular school
   - What type of institution is it? Public Private

5. In which class are you:
   - in 6th
   - in 5th
   - in 4th
   - in 3rd
   - in first year of CAP
   - in second year of CAP
   - in 2nd
   - in 1st

6. Since you go to school, have you ever repeated a grade? Yes No

7. Do you have private lessons at home? Yes No

8. Do you have personalized assistance at school, i.e. someone to help you in specific activities? Yes No

2. Medical history

1. Specialist follow-up in the past 12 months? Yes No
If yes:
- Pulmonologist
- Gastro-enterologist
- Neurologist
- Orthopedist
- Other

2. In the past 12 months, have you had any physiotherapy sessions? Yes No
If yes,
  - Respiratory physiotherapy
  - Motor physiotherapy

3. In the past 12 months, have you had any psychomotricity sessions? Yes No

4. In the past 12 months, have you had any session with a speech therapist? Yes No

5. In the past 12 months, did you go to a psychologist, psychiatrist? Yes No

6. Number of hospital admissions in the last 5 years: …..

7. Number of hospital admissions for respiratory cause in the last 5 years: ….

3. Family lifestyle

1. Where do you live most of the time?
  - With your mother and father
  - With your mother
  - With your father

2. Number of older siblings : ….

3. Number of younger siblings : ….

4. Number of stepsiblings: ….

5. Does your mother currently have a job?
  - Yes, full time
  - Yes, part time
  - No, she’s looking for a job
  - No, she takes care of the house
  - No, other reason

6. Does your father currently have a job?
  - Yes, full time

- Yes, part time
- No, she’s looking for a job
- No, she takes care of the house
- No, other reason
