# Supplementary material for: Educational and health outcomes associated with bronchopulmonary dysplasia in 15-year-olds born preterm
Source: PLoS One. 2019 Sep 11;14(9):e0222286. doi: 10.1371/journal.pone.0222286 (PMC6738652; doi:10.1371/journal.pone.0222286)
Supplement: S1 Table — (PDF) [file pone.0222286.s003.pdf]

S1 Table: Characteristics used for adjustment for each outcome studied.

Academic outcomes	Healthcare use	Family structure	Parental employment
Sex<br>Gestational age (GA)<br>Small for GA<br>Intraventricular hemorrhage<br>Periventricular leukomalacia<br>Necrotizing enterocolitis<br>Late onset sepsis<br>Post-natal steroids<br>Family structure<br>Maternal level of education<br>Socioeconomic status	Sex<br>Gestational age (GA)<br>Small for GA<br>Intraventricular hemorrhage<br>Periventricular leukomalacia<br>Necrotizing enterocolitis	Sex<br>Gestational age (GA)<br>Small for GA<br>Intraventricular hemorrhage<br>Periventricular leukomalacia<br>Specialist follow-up	Sex<br>Gestational age (GA)<br>Small for GA<br>Intraventricular hemorrhage<br>Periventricular leukomalacia<br>Specialist follow-up<br><br>Family structure<br>Number of siblings<br>Socio-economic status
