# Supplementary material for: Educational and health outcomes associated with bronchopulmonary dysplasia in 15-year-olds born preterm
Source: PLoS One. 2019 Sep 11;14(9):e0222286. doi: 10.1371/journal.pone.0222286 (PMC6738652; doi:10.1371/journal.pone.0222286)
Supplement: S2 Table — (PDF) [file pone.0222286.s004.pdf]

S2 Table: Comparison of academic and health outcomes of adolescents born very preterm with and without BPD, adjusted on parameters presented in Table 3 and retinopathy of prematurity.

	Ex-preterms with BPD (n=55)	Ex-preterms without BPD (n=249)	Adjusted OR (95% CI)	Adjusted p value
**Academic outcomes**				
School adapted for children with special needs or specialized institution (vs ordinary school)	14/55 (25%)	15/248 (6%)	9.9 (1.5-67.0)	0.02
Repeated grade	23/54 (43%)	52/246 (21%)	2.7 (0.9-8.0)	0.07
Personalized assistance at school	11/55 (20%)	9/247 (4%)	3.0 (0.4-22.7)	0.30
**Healthcare use**				
Specialist follow-up in the past 12 months	19/55 (35%)	61/249 (25%)	1.6 (0.7-3.6)	0.30
Physiotherapy in the last 12 months	10/55 (18%)	47/248 (19%)	0.3 (0.1-1.1)	0.08
Psychomotor therapist in the past 12 months	6/55 (11%)	2/248 (1%)	11.1 (0.9-145)	0.07
Speech therapist in the past 12 months	13/55 (24%)	15/248 (6%)	5.8 (1.6-21.2)	<0.01
Psychologist or psychiatrist in the past 12 months	15/55 (27%)	33/248 (13%)	2.0 (0.7-5.6)	0.20
≥ 1 hospital admission in the past 5 years	12/55 (22%)	44/248 (18%)	0.8 (0.3-2.3)	0.73

BPD: bronchopulmonary dysplasia

100 observations were deleted during these analyses due to missing values for the response or explanatory variables
