# Supplementary material for: Educational and health outcomes associated with bronchopulmonary dysplasia in 15-year-olds born preterm
Source: PLoS One. 2019 Sep 11;14(9):e0222286. doi: 10.1371/journal.pone.0222286 (PMC6738652; doi:10.1371/journal.pone.0222286)
Supplement: S3 Table — (PDF) [file pone.0222286.s005.pdf]

S3 Table: Characteristics of adolescents born very preterm included and lost to follow-up

	Included n=304 (%)	Lost to follow-up n=501 (%)	p
Sex			
▪ Male	154/304 (51%)	246/501 (49%)	0.72
▪ Female	150/304 (49%)	255/501 (51%)	
Gestational age[a]	30 (28-31)	30 (28-31)	0.11
Birth weight[a]	1250 (950-1600)	1310 (1010-1620)	0.25
▪ < 1000 g	89/304 (29%)	117/501 (23%)	0.17
▪ 1000-1499 g	115/304 (38%)	206/491 (41%)	
▪ ≥ 1500 g	100/304 (33%)	178/491 (35%)	
Small for gestational age	24/304 (8%)	38/491 (8%)	0.89
Apgar score at 1 min ≤ 3	69/287 (24%)	86/452 (19%)	0.12
Spontaneous prematurity	159/298 (53%)	288/482 (60%)	0.09
Bronchopulmonary dysplasia	55/304 (18%)	69/491 (14%)	0.13
Patent ductus arteriosus	11/302 (4%)	23/494 (5%)	0.59
Intraventricular hemorrhage	75/303 (25%)	113/495 (23%)	0.55
Periventricular leukomalacia	29/287 (10%)	54/471 (11%)	0.63
Necrotizing enterocolitis	7/303 (2%)	8/490 (2%)	0.59
Confirmed maternofetal infection	27/295 (9%)	32/487 (7%)	0.21
≥ 1 Sepsis	99/302 (33%)	152/492 (31%)	0.58
Post-natal steroids	89/302 (29%)	121/494 (24%)	0.14
SES of family			
▪ Professional	70/302 (23%)	94/498 (19%)	<0.01
▪ Intermediate	101/302 (33%)	117/498 (23%)	
▪ Administrative/public service, self-employed, student	78/302 (26%)	125/498 (25%)	
▪ Shop-assistant, service worker	31/302 (10%)	88/498 (18%)	
▪ Manual worker or unemployed	22/302 (7%)	74/498 (15%)	
Maternal age at birth			
▪ < 25	38/299 (13%)	75/495 (15%)	0.15
▪ 25-34	191/299 (64%)	331/495 (67%)	
▪ ≥ 35	70/299 (23%)	89/495 (18%)	

Parity at birth			
▪ 0	180/302 (60%)	269/493 (55%)	0.22
▪ 1-2	106/302 (35%)	185/493 (37%)	
▪ ≥ 3	16/302 (5%)	39/493 (8%)	
Maternal level of education			
▪ University	126/299 (42%)	153/462 (32%)	<0.01
▪ Secondary school 2nd part	82/299 (27%)	89/462 (19%)	
▪ Secondary school 1st part	77/299 (26%)	199/462 (42%)	
▪ Primary school or no school	14/299 (5%)	31/462 (7%)	
Country of birth of mother			
▪ France	249/300 (83%)	360/482 (75%)	<0.01
▪ Other	51/300 (17%)	122/482 (25%)	

a: results are presented as median with their interquartile 1-3. SES: socioeconomic status.
