# Supplementary material for: Educational and health outcomes associated with bronchopulmonary dysplasia in 15-year-olds born preterm
Source: PLoS One. 2019 Sep 11;14(9):e0222286. doi: 10.1371/journal.pone.0222286 (PMC6738652; doi:10.1371/journal.pone.0222286)
Supplement: S5 Table — (PDF) [file pone.0222286.s007.pdf]

S5 Table : Healthcare use in adolescents born very preterm with and without BPD

	All	Preterms with BPD (n=55)	Preterms without BPD (n=249)	p
Specialist follow-up in the past 12 months	80/304 (26%)	19/55 (35%)	61/249 (25%)	0.13
▪ Pulmonologist	20/304 (7%)	8/55 (15%)	12/249 (5%)	0.01
▪ Neurologist	12/304 (4%)	4/55 (7%)	8/249 (3%)	0.24
▪ Gastroenterologist	3/304 (1%)	1/55 (2%)	2/249 (1%)	0.45
▪ Orthopedist	20/304 (7%)	3/55 (5%)	17/249 (7%)	1.00
▪ Other	43/304 (14%)	9/55 (16%)	34/249 (14%)	0.27
Physiotherapy in the last 12 months	57/303 (19%)	10/55 (18%)	47/248 (19%)	0.89
▪ Chest physiotherapy	3/303 (1%)	2/55 (4%)	1/248 (0.4%)	0.03
▪ Motor physiotherapy	54/303 (18%)	8/55 (15%)	46/248 (19%)	0.48
Psychomotor therapist in the past 12 months	8/303 (3%)	6/55 (11%)	2/248 (1%)	<0.01
Speech therapist in the past 12 months	28/303 (9%)	13/55 (24%)	15/248 (6%)	<0.01
Psychologist or psychiatrist in the past 12 months	48/303 (16%)	15/55 (27%)	33/248 (13%)	0.01
≥ 1 hospital admission in the past 5 years	56/303 (18%)	12/55 (22%)	44/248 (18%)	0.48
▪ Hospital admission for respiratory condition	0	0	0	

BPD: bronchopulmonary dysplasia

erquartile 1-3. SES: socioeconomic status.
