# Supplementary material for: Educational and health outcomes associated with bronchopulmonary dysplasia in 15-year-olds born preterm
Source: PLoS One. 2019 Sep 11;14(9):e0222286. doi: 10.1371/journal.pone.0222286 (PMC6738652; doi:10.1371/journal.pone.0222286)
Supplement: S6 Table — (PDF) [file pone.0222286.s008.pdf]

S6 Table: Family characteristics of adolescents born very preterm with and without BPD

	All	Preterms with BPD (n=55)	Preterms without BPD (n=249)	p
Family structure				
▪ Two-parent family	243/303 (80%)	46/55 (84%)	197/248 (79%)	0.67
▪ Single-parent family	58/303 (19%)	9/55 (16%)	49/248 (20%)	
▪ Foster care, institution	2/303 (1%)	0/55 (0%)	2/248 (1%)	
Siblings				
0	88/303 (29%)	12/55 (22%)	76/248 (31%)	0.17
1	124/303 (41%)	20/55 (36%)	104/248 (42%)	
2	67/303 (22%)	16/55 (29%)	51/248 (21%)	
≥3	24/303 (8%)	7/55 (13%)	17/248 (7%)	
Older siblings	113/303 (37%)	22/55 (40%)	91/248 (37%)	0.37
Younger siblings	130/303 (43%)	26/55 (47%)	104/248 (42%)	0.11
Mother’s employment status				
Employed	240/303 (79%)	45/55 (82%)	195/248 (79%)	0.60
▪ Full-time job	155/303 (51%)	25/55 (45%)	130/248 (52%)	0.32
▪ Part-time job	85/303 (28%)	20/55 (36%)	65/248 (26%)	
Unemployed	63/303 (21%)	10/55 (18%)	53/248 (21%)	
Father’s employment status				
Employed	260/297 (88%)	46/53 (87%)	214/244 (88%)	0.86
▪ Full-time job	255/297 (86%)	45/53 (85%)	210/244 (86%)	0.97
▪ Part-time job	5/297 (2%)	1/53 (2%)	4/248 (2%)	
Unemployed	37/297 (12%)	7/53 (13%)	30/244 (12%)	

BPD: bronchopulmonary dysplasia
